# Polyethylene wear of dual mobility cups: a comparative analysis based on patient-specific finite element modeling

**DOI:** 10.1007/s00264-022-05305-4

**Published:** 2022-01-12

**Authors:** Julien Wegrzyn, Alexander Antoniadis, Ehsan Sarshari, Matthieu Boubat, Alexandre Terrier

**Affiliations:** 1 Department of Orthopedic Surgery, Lausanne University Hospital—Centre Hospitalier Universitaire Vaudois—CHUV, Hôpital Orthopédique, Avenue Pierre-Decker, 4, CH-1011 Lausanne, Switzerland; 2 Laboratory of Biomechanical Orthopedics, Ecole Polytechnique Fédérale de Lausanne, Lausanne, Switzerland

**Keywords:** Dual mobility cup, Polyethylene mobile component, Patient-specific modeling, Polyethylene wear, Highly cross-linked polyethylene

## Abstract

**Purpose:**

Concerns remain about potential increased wear with dual mobility cups related to the multiple articulations involved in this specific design of implant. This finite element analysis study aimed to compare polyethylene (PE) wear between dual mobility cup and conventional acetabular component, and between the use of conventional ultra-high molecular weight PE (UHMWPE) and highly cross-linked PE (XPLE).

**Methods:**

Patient-specific finite element modeling was developed for 15 patients undergoing primary total hip arthroplasty (THA). Five acetabular components were 3D modeled and compared in THA constructs replicating existing implants: a dual mobility cup with a 22.2-mm-diameter femoral head against UHMWPE or XLPE (DM22PE or DM22XL), a conventional cup with a 22.2-mm-diameter femoral head against UHMWPE (SD22PE) and a conventional cup with a 32-mm-diameter femoral head against UHMWPE or XLPE (SD32PE or SD32XL).

**Results:**

DM22PE produced 4.6 times and 5.1 times more volumetric wear than SD32XL and DM22XL (*p* < 0.0001, Cohen’s *d* = 6.97 and 7.11; respectively). However, even if significant, the differences in volumetric wear between DM22XL and SD32XL as well as between DM22PE and SD22PE or SD32PE were small according to their effect size (*p* < 0.0001, Cohen’s |*d*|= 0.48 to 0.65) and could be therefore considered as clinically negligible.

**Conclusion:**

When using XLPE instead of UHMWPE, dual mobility cup with a 22.2-mm-diameter femoral head produced a similar amount of volumetric wear than conventional acetabular component with a 32-mm-diameter femoral head against XLPE. Therefore, XLPE is advocated in dual mobility cup to improve its wear performance.

## Introduction

Although dual mobility cups demonstrate a marked increase in use to achieve hip stability during primary and revision total hip arthroplasty (THA), concerns remain about potential increased wear related to the multiple articulations involved in this specific design of implant [1–4]. Indeed, the polyethylene (PE) mobile component is involved in three prosthetic articulations [5–7]. At the small articulation, the concave inner bearing surface articulates with the femoral head and behaves as a low-friction bearing [5–7]. At the large articulation, the convex outer bearing surface articulates against the metal shell and behaves as a large effective PE-head bearing that increases the jump distance to dislocation [5–7]. The impingement-free range of motion within a dual mobility cup is also increased compared to a conventional bearing due to the third articulation that engages movement of the PE mobile component at the large articulation upon femoral neck contact onto the chamfer [5–7]. Consequently, the convex outer bearing surface of the PE mobile component was supposed to introduce an additional source of PE wear [4]. However, previous retrieval and biomechanical studies demonstrated that motion and wear within dual mobility cup predominate at the small articulation in vivo [6–11]. Nevertheless, their major limitations are related to the fact that retrieval studies are non-comparative and evaluate wear performance on explants potentially issued from THA with non-optimal functioning in vivo and/or PE damage to the mobile component that could occur at the time of revision [9–11]. In addition, biomechanical studies using hip simulator with gravimetric measurement usually do not consider parameters such as patient’s level of activity and hip anatomy, body mass index (BMI) or variation in implant positioning related to surgeon’s experience [6–8]. Another limitation of these ex vivo studies is related to their ability to determine the exact parts played in wear by the small and large articulations [6–11].

Therefore, there is a need for a better understanding, quantification and prediction of wear of dual mobility cup. In previous studies [12–14], we developed patient-specific modeling of the hip using finite element analysis (FEA) to simulate the in vivo biomechanical functioning of THA that enables simulation and evaluation of wear of dual mobility and conventional cups. This FEA study aimed to evaluate and compare PE wear between dual mobility and conventional cups, as well as between the use of conventional ultra-high molecular weight PE (UHMWPE) or highly cross-linked PE (XPLE).

## Material and methods

### Patients

**Fig. 1:**
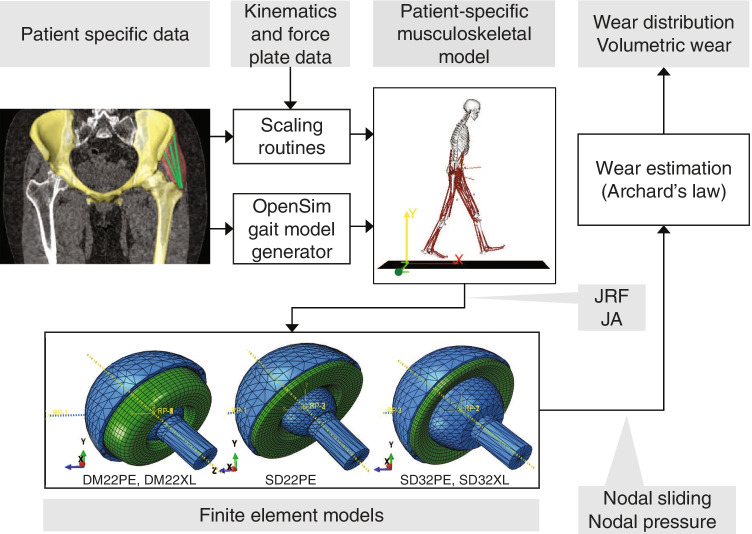
Workflow of the patient-specific modeling using patient’s hip anatomy, weight and height to predict joint reaction force (JRF) and joint angle (JA), which were used as boundary conditions of the finite element models of dual mobility (DM22PE and DM22XL) and conventional (SD22PE, SD32PE and SD32XL) cup constructs to evaluate polyethylene wear

Patient-specific finite element modeling was developed for 15 patients (eight men, mean age = 50 ± 14 years, mean BMI = 30 ± 4 kg/m^2^). The inclusion criteria were patients undergoing primary THA for hip osteoarthritis at our institution and having a preoperative computed-tomography (CT) scan of the entire pelvis and hip with a sufficient resolution to build computer models. The only exclusion criteria (to avoid bias) were to balance the gender ratio and to account for a large range of age and anatomic variability, especially regarding the femoral offset. These patient-specific models were described with more details in previous studies [12–14]. For each patient, 3D geometric models of the pelvis and femur were developed by segmentation of preoperative CT scan. Then, THA implants were replicated in these models. A generic musculoskeletal model was adapted to each patient anatomy. This model was used to predict hip kinematics and joint reaction force during level walking that were used as boundary conditions to predict wear of PE components in a FEA model (Fig. 1). Patient’s informed consent and Institutional Review Board approval were obtained before initiating this study (CER-VD#2013).

### THA implants

For each virtual patient, five acetabular components with a 52-mm external diameter were 3D modeled and compared in THA constructs replicating existing implants (Symbol®, Dedienne santé, Mauguio, France): a dual mobility cup with a 22.2-mm-diameter femoral head against UHMWPE (DM22PE), a conventional cup with a 22.2-mm-diameter femoral head against UHMWPE (SD22PE), a conventional cup with a 32-mm-diameter femoral head against UHMWPE (SD32PE), a conventional cup with a 32-mm-diameter femoral head against XLPE (SD32XL) and a dual mobility cup with a 22.2-m- diameter femoral head against XLPE (DM22XL) (Fig. 1). A same design of femoral stem (Symbol®) was used with a simulated cobalt-chromium head.

### Musculoskeletal and finite element model

**Fig. 2:**
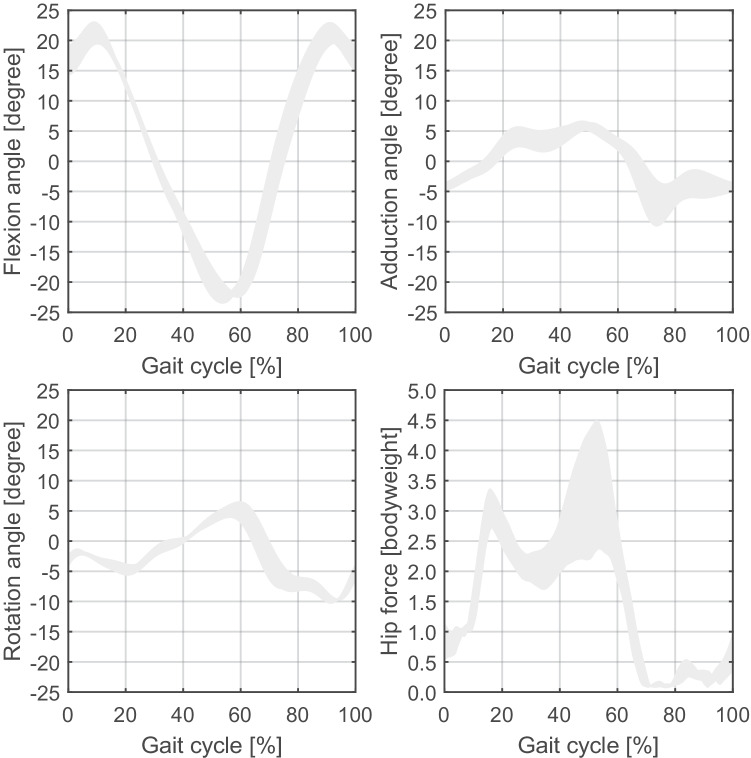
Hip angles and joint reaction force during the walking cycle. The gray area represents the minimum and maximum values over the 15 patients

A generic (inverse dynamics) OpenSim lower limb musculoskeletal model (Model Gait 2392) was used to predict hip kinematics and joint reaction force during level walking [15] (Figs. 1 and 2). This model was adapted to the hip anatomy of each patient (i.e. rotation centre and origin of the 3 gluteus muscles) that was estimated from the pre-operative CT scan as well as to their height and weight [14]. The rotation centre of the operated hip was evaluated by fitting a sphere into the acetabulum surface. Then, eleven level walking cycles of the gait model were simulated and averaged to define a single level walking cycle per patient. A Matlab script (MathWorks Inc, Natick, MA) was developed to automatically adapt the generic model using the OpenSim Application Programming Interface.

A finite element model was developed in Abaqus (3DS Dassault Systèmes, Vélizy-Villacoublay, France) for each THA implant [16]. The metal shell was placed first with 40° of inclination and 15° of anteversion with respect to the pelvic anatomical landmarks. For UHMWPE components, the elasto-plastic material properties were elastic modulus of 500 MPa, Poisson’s ratio of 0.45 and plastic yield stress of 16 MPa [17]. For annealed XLPE components, the elasto-plastic material properties were elastic modulus of 1000 MPa, Poisson’s ratio of 0.45 and plastic yield stress of 20 MPa [18]. Bone and metal structures were assumed rigid and modeled as analytical surfaces while PE was modeled as linear elastic. Linear hexahedral elements were then used for modeling the PE components. For both conventional and dual mobility cups, a contact was considered between the PE component and the femoral head. The PE mobile component was allowed to move freely with respect to the metal shell in dual mobility cup. Contrarily, the PE component was fully constrained as an insert into the metal shell in conventional cup. We used the same coefficient of friction (*µ* = 0.01) for all metal-PE interfaces [19]. The boundary conditions of the finite element models were defined to reproduce the kinematics and joint reaction force predicted by the musculoskeletal model during the entire walking gait cycle. The variable rotation of the femoral head was imposed by a connector element in Abaqus, while the varying joint reaction force vector was applied in parallel on the femoral head center. The metal shells were fixed. The same procedure was performed for each of the 15 patients and five cups.

### Wear model

The Archard wear law was used to predict PE wear from contact pressure and sliding distance [20]. For UHMWPE, the wear coefficient was: 10.656 × 10^−7^ mm^3^ N^−1^ m^−1^ [21]. For annealed XLPE, 20% of the UHMWPE wear coefficient was considered [22]. For dual mobility cup, wear of the convex outer (“large articulation”) and concave inner (“small articulation”) bearing surfaces of the PE mobile component was considered. Pressure and sliding of the femoral head onto the PE component were predicted by the FEA model. A Python routine function was developed to define linear wear on each finite element node of the PE surface according to the Archard wear equation, and extrapolated to 1.0 million cycles (mc) of level walking gait that corresponds approximately to one year of normal activity [23]. Testing to 1.0 mc aimed to imitate the in vivo steady-state wear [6, 7, 23, 24]. The steady-state wear of PE bearing is mathematically linear as a function of cycle count, making wear highly predictable over 1.0 mc [6, 7, 23, 24]. Volumetric wear was derived from linear wear and corresponded to the difference between the initial and final volume of the PE component. Linear and volumetric wear values were reported in mm and mm^3^, respectively. A mesh convergence analysis was performed with the volumetric wear of DM22PE. We evaluated five mesh refinements to reach a variability lower than 1.5%, using an average mesh size of 0.75 mm and resulting in about 400,000° of freedom. The model was indirectly validated by comparison with similar numerical and experimental studies on comparable SD22PE designs [21].

### Statistical analysis

Descriptive statistics were reported as mean ± standard deviation (SD). Normality of all variables was tested using Shapiro–Wilk test. DM22PE was considered as the control group for linear and volumetric wear comparisons. Comparison between two quantitative variables was performed using two-sided paired *t* tests and reported with mean ± SD and effect size (Cohen’s *d*) of the difference. The 95% confidence interval (95% CI) was reported for the difference and effect size. Relationship between two quantitative variables was assessed using the coefficient of determination (*R*^2^). Statistical analyses were performed with R 4.0 software (cran.r-project.org) with a level of significance set at *p* < 0.05.

## Results

**Fig. 3:**
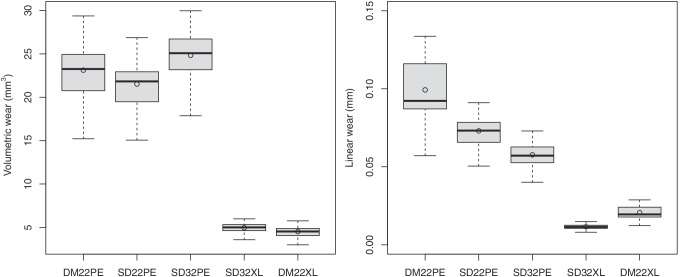
Volumetric (**A**) and linear (**B**) wear rates of polyethylene components for dual mobility cup with a 22.2-mm-diameter femoral head against UHMWPE (DM22PE), conventional cup with a 22.2-mm-diameter femoral head against UHMWPE (SD22PE), conventional cup with a 32-mm-diameter femoral head against UHMWPE (SD32PE), conventional cup with a 32-mm-diameter femoral head against highly cross-linked PE (SD32XL) and dual mobility cup with a 22.2-mm-diameter femoral head against highly cross-linked PE (DM22XL). The boxplots show quartiles and mean (circle)

Volumetric and linear wear rates of DM22PE, SD22PE, SD32PE, SD32XL and DM22XL are reported in Table 1 and Fig. 3.

**Table 1:** Volumetric and linear wear rates of the 5 acetabular components, and differences with the DM22PE control group (DM22PE or DM22XL: dual mobility cup with a 22.2-mm-diameter femoral head against UHMWPE or highly cross-linked polyethylene [XLPE], SD22PE: conventional cup with a 22.2-mm-diameter femoral head against UHMWPE, SD32PE or SD32XL: conventional cup with a 32-mm-diameter femoral head against UHMWPE or XLPE, d: effect size, 1.0 mc: 1 million cycles of simulated level walking gait)

		Difference with DM22PE		
	Mean ± SD	Mean ± SD [95% CI]	*d* [95% CI]	*p*
Volumetric wear (mm^3^ at 1.0 mc)				
DM22PE (control group)	23.1 ± 3.6			
SD22PE	21.5 ± 3.0	1.6 ± 0.8 [1.2, 2.0]	0.48 [− 0.25,1.20]	< 0.0001
SD32PE	24.8 ± 3.1	− 1.7 ± 1.1 [− 2.3, − 1.1]	− 0.51 [− 1.23, 0.23]	< 0.0001
SD32XL	5.0 ± 0.7	18.2 ± 3.0 [16.5, 19.9]	6.97 [5.01, 8.81]	< 0.0001
DM22XL	4.5 ± 0.7	18.6 ± 2.9 [17.0, 20.2]	7.11 [5.11, 9.08]	< 0.0001
Linear wear (mm at 1.0 mc)				
DM22PE (control group)	0.099 ± 0.023			
SD22PE	0.073 ± 0.011	0.026 ± 0.014 [0.019, 0.034]	1.47 [0.64, 2.27]	< 0.0001
SD32PE	0.058 ± 0.009	0.042 ± 0.015 [0.033, 0.050]	2.42 [1.45, 3.36]	< 0.0001
SD32XL	0.012 ± 0.002	0.088 ± 0.021 [0.076, 0.099]	5.43 [3.83, 7.00]	< 0.0001
DM22XL	0.020 ± 0.005	0.079 ± 0.018 [0.069, 0.089]	4.77 [3.33, 6.20]	< 0.0001

### Volumetric wear

**Fig. 4:**
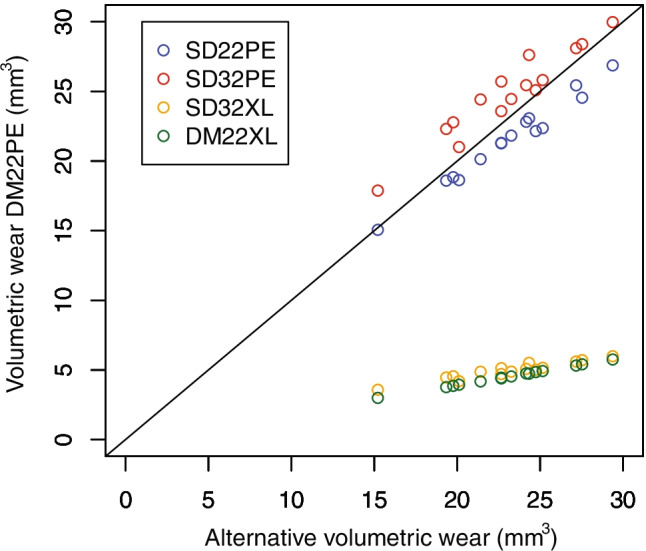
Volumetric wear of SD22PE, SD32PE and DM22XL (y-axis) compared to DM22PE (x-axis) for the 15 simulated patients

DM22PE produced 1.1 times more volumetric wear than SD22PE, 4.6 times more volumetric wear than SD32XL and 5.1 times more volumetric wear than DM22XL (*p* < 0.0001). However, DM22PE produced 0.9 times less volumetric wear than SD32PE (*p* < 0.0001). In addition, DM22XL produced 0.9 times less volumetric wear than SD32XL (difference =  − 0.44 ± 0.22 mm^3^, 95% CI = [− 0.56, − 0.31]; *d* =  − 0.65, 95% CI = [− 1.39, 0.09]; *p* < 0.0001). However, even if significant, the differences in volumetric wear between DM22PE and SD22PE or SD32PE and between DM22XL and SD32XLwere small according to their effect size and could be therefore considered as clinically negligible (Fig. 4).

**Fig. 5:**
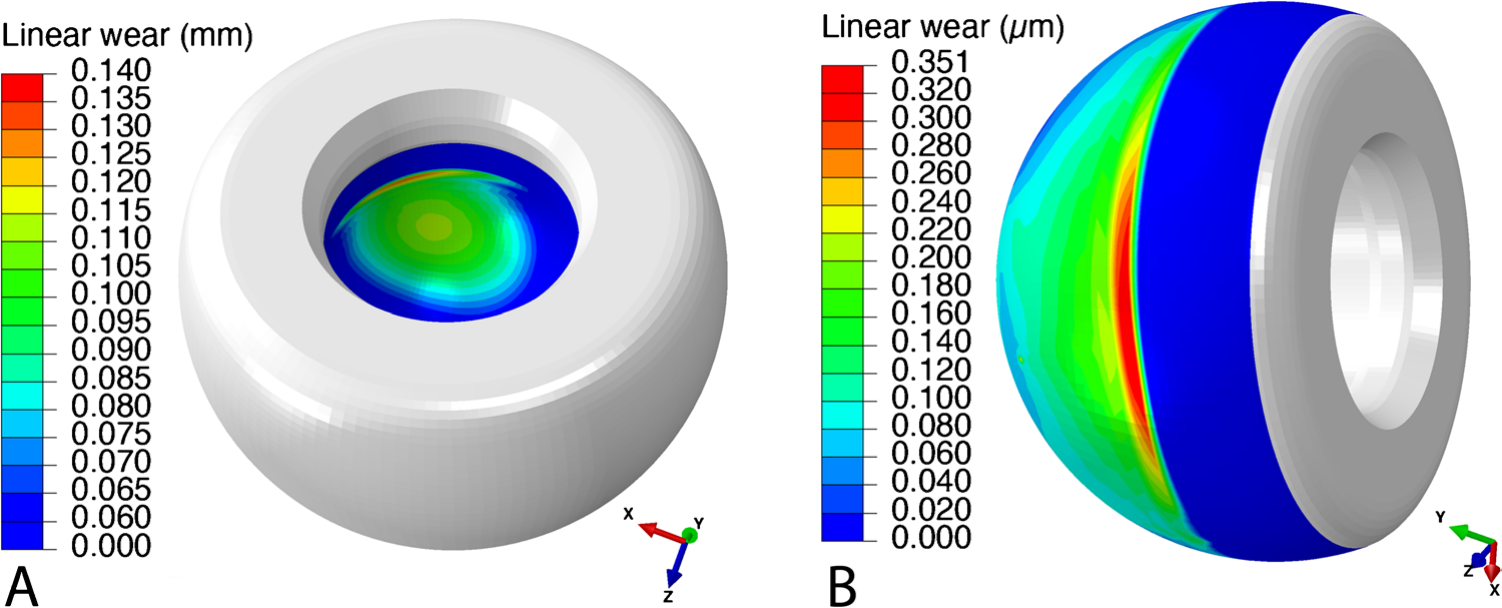
Wear repartition onto the concave inner (**A**) and convex outer (**B**) bearing surfaces of the polyethylene mobile component (DM22PE). Note: the color scales are different

Regarding the repartition of wear during the simulated gait cycle, the volumetric wear of the convex outer bearing surface at the large articulation was 0.13 ± 0.03 mm^3^ for DM22PE and 0.01 ± 0.003 mm^3^ for DM22XL, whereas the volumetric wear of the concave inner bearing surface at the small articulation was 23.0 ± 3.6 mm^3^ and 4.5 ± 0.7 mm^3^, respectively (Fig. 5).

**Fig. 6:**
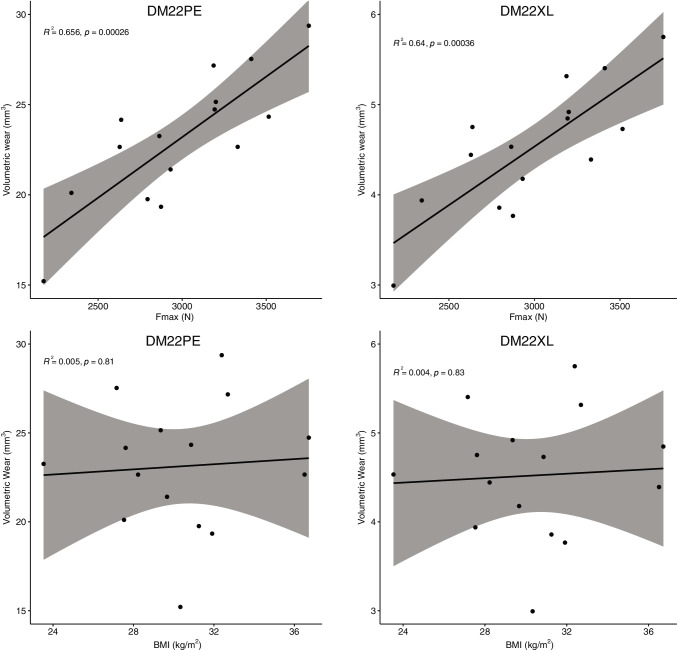
Correlations between the volumetric wear of DM22PE and DM22XL and maximum joint reaction force (**A**) and BMI (**B**) for the 15 simulated patients (gray area represent 95% CI)

For both DM22PE and DM22XL, the volumetric wear was positively correlated to joint reaction force (*R*^2^ = 0.656 and 0.640; *p* = 0.0003 and 0.0004), but not to BMI (*R*^2^ = 0.005 and 0.004; *p* = 0.81 and 0.83) (Fig. 6).

### Linear wear

The linear wear of DM22PE was significantly higher than of SD22PE, SD32PE, SD32XL and DM22XL (*p* < 0.0001). In addition, the linear wear of DM22XLPE was significantly higher than of SD32XL (difference = 0.009 ± 0.003 mm, 95% CI = [0.007, 0.010]; *d* = 2.47, 95%-CI = [1.49, 3.42]; *p* < 0.0001).

## Discussion

Although reports on the long-term wear in vivo of recent designs of dual mobility cup are sparse in literature, previous clinical and laboratory studies suggest that wear of dual mobility cup compared favorably with conventional PE bearings with large heads routinely used in THA [1–3]. For most of the modern designs of dual mobility cup, the mobile component is made of UHMWPE with survival rates reported up to 93% at ten years, 84% at 15 years and 74% at 22 years in primary THA [5, 25, 26]. As contemporary THA patients are more active and functionally demanding than patients evaluated in historical series, the use of XLPE for the mobile component was introduced in dual mobility cup to increase wear performance [27]. A recent analysis of the American Joint Replacement Registry further reported that patients undergoing primary THA under the age of 50 years demonstrated the highest rates of dual mobility cup utilization [2]. However, Boyer et al. [26] reported that patients of this specific age group presented higher risk of dual mobility cup revision for wear and aseptic loosening at long-term follow-up compared to patients over 60 years of age. Therefore, this current trend in the use of dual mobility cup emphasizes the need for a better understanding, quantification and prediction of wear in order to optimize both indication of this bearing and material selection for the PE mobile component. The most important finding of this study was that dual mobility cup with a mobile component made of UHMWPE produced significantly higher volumetric wear than conventional cup with a 32-mm-diameter head articulating against XLPE or a dual mobility cup with a mobile component made of XLPE. Importantly, a similar amount of volumetric wear could be expected between dual mobility cup with a mobile component made of XLPE and conventional cup with a 32-mm-diameter head articulating against XLPE.

To our knowledge, this study based on patient-specific finite element modeling is the first to evaluate and compare wear between dual mobility and conventional cups with PE components made of UHMWPE or XLPE. Several attempts at measuring PE wear of the outer convex and inner concave bearing surfaces of mobile components were performed through clinical, retrieval or hip joint simulator studies [3, 6–11]. However, no standardized method of quantifying wear of dual mobility cup has been reported in literature. Regarding the ex vivo studies, the quantification of wear for PE bearings could be overestimated in retrieval studies while hip simulator studies would tend to underestimate it [24, 28]. In retrieval studies, Adam et al. and Geringer et al. [10, 11] both demonstrated a volumetric wear rate of 54 mm^3^/year in dual mobility cup mobile components made of UHMWPE that was higher than the rate of 24 mm^3^ we observed in our numerical study after a simulated one year of level walking. Comparable to our results, Saikko et al. [8] reported, in a comparative hip joint simulator study, a similar amount of volumetric wear between dual mobility and conventional cups with PE components made of UHMWPE. Moreover, Saikko et al. [8] demonstrated that machining marks were almost intact at the convex outer bearing surface of mobile components suggesting that motion at the large articulation was minimal. Similarly, in a retrieval study evaluating PE damage and wear lesions to the bearing surfaces of mobile components, D’Apuzzo et al. [9] showed that, although occurring at both bearing surfaces, motion and wear within a dual mobility cup predominate at the small articulation. In our study, motion and volumetric wear predominated at the small articulation for both DM22PE and DM22XL. Importantly, the ratio of volumetric wear of the convex outer bearing surface to the concave inner bearing surface was 1/187 for DM22PE and 1/353 for DM22XL. Therefore, contrarily to the concern raised by Deckard et al. [4] about potential increased wear due to the large articulation, the convex outer bearing surface of the PE mobile component did not represent a significant additional source of wear with dual mobility cup. Besides these laboratory studies, Boyer et al. [29] reported that wear is tridimensional in vivo as the PE mobile component is intended to move freely around three axes. Therefore, measurement of the linear penetration rate on conventional two-dimensional radiographs is deemed not to be effective for determining wear of dual mobility cup [29]. Therefore, some authors proposed the use of 3D radiostereometric analysis (RSA) to measure wear in vivo though no correlation with explant analysis was performed to confirm the accuracy of this technique [30]. In our study, for both DM22PE and DM22XL, the linear wear reported was below the 0.1 mm/year threshold that was proposed by Dumbleton et al. [31] to increase the risk of osteolysis. In addition, the rate of linear wear predicted in our study with DM22XL was equivalent to the rate measured by Laende et al. [30] with 3D RSA technique to evaluate dual mobility cup with XLPE mobile component in vivo. This same linear wear rate of 0.020 mm/year was within the expected range of wear for conventional cup with large head against XLPE [30, 32]. Moreover, similarly to this 3D RSA study, we found that wear of dual mobility cup was independent from patient BMI for both DM22PE and DM22XL suggesting that dual mobility cup could be used in high BMI patients without increased risk of wear compared to conventional acetabular components.

This numerical study presented with some limitations. Wear varies from patient to patient, even in the case of identical implants, being influenced by numerous factors such as patient’s activity, and quality of bone, muscle and hip reconstruction, which is difficult to reproduce and control ex vivo. In the present study, we tried to account for this variability using patient-specific modeling issued from 15 different patients. In addition, only level walking was replicated, instead of more complex and demanding movements of daily living activities such as stair negotiation or running. This generic level walking is certainly the most important limitation of our study. In addition, we are obviously limited by a purely computational study that cannot reproduce the entire complexity of the post-operative reality. We might certainly have predicted higher wear at the convex outer surface of the dual mobility mobile components. We might also have observed more wear at the third articulation, which was virtual in the present study. Finally, this numerical study only replicated 1.0 mc of level walking, corresponding approximately to one year of normal activity after THA. Extending this postoperative time would have required more complex modeling. Besides, we considered here a constant wear coefficient, while it has been reported to be pressure dependent and multidirectional [33, 34]. However, for sake of simplicity, we assumed that it would not affect this comparative study. We thus believe that these limitations would not have changed our main conclusions.

## Conclusion

This numerical study based on patient-specific finite element modeling demonstrated that, when using XLPE instead of UHMWPE, dual mobility cup with a 22.2-mm-diameter femoral head produced a similar amount of volumetric wear than conventional cup with a 32-mm-diameter femoral head articulating against XLPE. Therefore, the use of XLPE is advocated in dual mobility cup to improve its wear performance especially in young, active and high functional demand patients.

## Data Availability

All the data and material are saved in an anonymized repository file folder and available upon request.
